# Implementing recommended breastfeeding practices in healthcare facilities in India during the COVID-19 pandemic: a scoping review of health system bottlenecks and potential solutions

**DOI:** 10.3389/fnut.2023.1142089

**Published:** 2023-07-31

**Authors:** Nonita Dudeja, Divita Sharma, Arti Maria, Priyanka Pawar, Ritika Mukherjee, Shikha Nargotra, Archisman Mohapatra

**Affiliations:** ^1^Generating Research Insights for Development (GRID) Council, Executive Office, Noida, Uttar Pradesh, India; ^2^Department of Neonatology, Atal Bihari Vajpayee Institute of Medical Sciences and Dr. Ram Manohar Lohia Hospital, New Delhi, India

**Keywords:** breastfeeding, COVID-19, maternal health, newborn care, hospital

## Abstract

**Background:**

Breastfeeding practices in institutional settings got disrupted during the COVID-19 pandemic. We reviewed the challenges faced and the “work-around” solutions identified for implementing recommended breastfeeding practices in institutionalized mother-newborn dyads in resource constrained settings during the pandemic with the aim to identify learnings that could be potentially adapted to the Indian and relatable contexts, for building resilient health systems.

**Methods:**

We conducted a scoping review of literature using the PRISMA ScR Extension guidelines. We searched the Medline via PubMed and Web of Science databases for literature published between 1st December 2019 and 15th April 2022. We included original research, reviews, and policy recommendations published in English language and on India while others were excluded. Further, we searched for relevant gray literature on Google (free word search), websites of government and major professional bodies in India. Three reviewers independently conducted screening and data extraction and the results were displayed in tabular form. Challenges and potential solutions for breastfeeding were identified and were categorized under one or more suitable headings based on the WHO building blocks for health systems.

**Results:**

We extracted data from 28 papers that were deemed eligible. Challenges were identified across all the six building blocks. Lack of standard guidelines for crisis management, separation of the newborn from the mother immediately after birth, inadequate logistics and resources for infection prevention and control, limited health workforce, extensive use of formula and alternative foods, inconsistent quality of care and breastfeeding support, poor awareness among beneficiaries about breastfeeding practices (and especially, about its safety during the pandemic) were some of the challenges identified. The solutions primarily focused on the development of standard guidelines and operating procedures, restricted use of formula, use of telemedicine services for counseling and awareness and improving resource availability for risk mitigation through strategic mobilization.

**Conclusion:**

The COVID-19 pandemic has provided rich learning opportunities for health system strengthening in India. Countries must strengthen learning mechanisms to identify and adapt best practices from within their health systems and from other relatable settings.

## 1. Introduction

Breastfeeding is one of the best known interventions to reduce neonatal and infant mortality (1). However, despite investment in efforts, the 2019 Global Breastfeeding Score Card by World Health Organization (WHO) and United Nations Children's Emergency Fund (UNICEF) reported that breastfeeding practices were universally in poor alignment with the recommendations, especially in resource constrained settings (2–5). The COVID-19 pandemic that hit in 2020 caused further disruption to routine institutional breastfeeding practices in India and other low and middle income countries, and the rates are likely to have suffered further (6, 7).

India has the largest annual birth cohort in the world; about 23 million babies are born in India every year which is almost equal to the annual birth cohort of the next three countries i.e., China, Nigeria and Pakistan taken together. There is strong commitment to improve breastfeeding practices in healthcare facilities in India. However, inconsistencies persist. As per the latest National Family Health Survey [NFHS-5 (2019–21); India's Demographic Health Survey], while 88.6% of the deliveries in India happen in institutional settings, only about 42% of mothers manage to breastfeed their newborn within an hour of delivery (early initiation of breastfeeding) (8). This suggests that there is much need for strengthening breastfeeding practices in institutionalized mother-newborn dyads (9–13). It must be acknowledged that the aforementioned statistics could be an oversimplification. While on one hand there are health facilities both in the government and in the private sector that have world-class practices and capacity, there are the ones that are compromised in terms of skill, capacity and motivation. Thus, India provides a rich context for health system learning that need not be limited to within-country consumption but of immense value for global debate and adaptation (14–16). In the past, learnings from India have influenced global health action for maternal and child health. For example, India was the first country to launch a family planning program. India's Anganwadi Program is the largest community-based program in the world for early childhood education and nutrition (17). Even for polio eradication and for strengthening newborn programs, insights from India have been leveraged by other countries (18, 19). As the largest member in the South East Asia Region, India has major influence in the region's health performance and policy decisions. Nevertheless, the Indian context continues to be heterogenous, resource inconsistent and complex with ample opportunity for improvement, standardization and cross-learning (20, 21).

The COVID-19 pandemic challenged the well-established practices of newborn care (22–24). At the beginning of the pandemic, the possibility of vertical or horizontal transmission was unknown, and it was not fully understood how neonates could be affected. Further, the pandemic posed challenges to the quality of care at health care institutions on various fronts including provisions for breastfeeding support to the mother and her newborn, kangaroo mother care (KMC), family participatory care, and human milk bank operations (6, 25, 26). Fears around the spread of COVID-19 infection, lack of resources, technical and infrastructural limitations and lack of coherent guidelines coincided with the simultaneous efforts toward protecting institutional practices for breastfeeding. Concerns revolved around practicing skin-to-skin contact (SSC), rooming-in and breastfeeding by COVID-19 suspected, probable or confirmed mothers.

In early 2020, WHO conducted a living systematic review, to identify studies including mothers with suspected or confirmed COVID-19 and their infants or young children, in order to assess the outcomes of COVID-19 infected mothers who were breastfeeding (27). It was found that the risk of COVID-19 infection was low and the infection was typically mild or asymptomatic in newborns, while the results of not breastfeeding and separation of mother and the newborn were significantly negative (27). Consequently, WHO recommended that mothers who were suspected, probable, or confirmed cases of COVID-19 should follow standard infant feeding practices along with necessary precautions for IPC. These recommendations included that for continuous and prolonged skin-to-skin contact immediately after birth, initiation of breastfeeding within 1 h of birth, rooming-in and exclusive breastfeeding. Mothers were advised to mandatorily take IPC precautions, including hand washing, using medical masks and cleaning of frequently touched surfaces (28). However, a survey of 33 countries conducted around that time showed that the guidelines in these countries were not in alignment with the recommendations from WHO (29). As knowledge accrued over the year 2020, many studies started reporting on neonatal outcomes and recommended approaches to handling of the mother-infant dyad during the COVID-19 pandemic. Gradually, countries' guidelines including those released by the Indian scientific bodies got in line with those from WHO (30, 31). Nevertheless, the chaos that prevailed in the meanwhile led to several work around solutions, some as innovations for resource optimization while many as temporary make-shift arrangements. These called for a careful compilation and gave an opportunity for critical learning.

This review was planned with a view to understand the breastfeeding related challenges in institutional settings during the COVID-19 pandemic and the approaches identified as measures to combat these challenges in resource constrained settings with India as a candidate example. The purpose was to collate scientific evidence on key interventions that may be useful in implementing recommended breastfeeding practices with resilience within institutional settings for mother-newborn dyads in India and relatable contexts in the event of a future pandemic/public health crisis.

## 2. Methods

The review followed the scoping review extension of Preferred Reporting Items for Systematic Review and Meta-Analysis (PRISMA-ScR) guidelines.

### 2.1. Data sources and search strategy

We searched Medline via PubMed and Web of Science databases for the combinations of four sets of keywords. The first set consisted of the words: “mother”, “female”, “woman”, “neonate”, “newborn”, “infant”, “nursing mother”. The second set included: “pandemic,” “COVID-19”, “Coronavirus”, “SARS CoV-2”, “severe acute respiratory syndrome coronavirus 2”. The third set included: “breastfeeding”, “breast feed”, “lactation”. The fourth set included: “institutionalized”, “hospitalized”, “maternity ward”, “nursery”. The words in each set were separated by an OR Boolean. We used all word variations and the searches were done in the title and abstract as well as MeSH word search. The fourth set of words were searched in all fields. In the final search, we used combination of words from the four sets by placing AND between the search sets (detailed search strategy is presented in Supplementary Table 1). The search was conducted for the time frame of 1 December 2019 to 15 April 2022. All study designs were included. We restricted the search to articles from India with an aim to collate information for the Indian context. Further, we searched for relevant gray literature using similar keywords (for e.g., mother, breastfeeding, lactation, COVID-19, hospital, etc.) as that of the search strategy on Google (free word search), Government of India website (www.nhm.gov.in) and on websites of major professional bodies in India i.e., IAP, NNF, FOGSI, BPNI, IAPSM, IPHA, WHO, and UNICEF; we looked for policy documents, guidelines and advisories.

### 2.2. Article selection and data extraction

Three researchers (ND, DS, SN) independently screened the title and abstracts of the documents identified through the search, followed by screening those short-listed by reviewing the full text. The conflicts were resolved after discussion and fourth reviewer's comments, if necessary. Thereafter, information relevant to the study objectives was extracted onto a pre-defined data extraction tool. Extracted information included details about the study characteristics (authors, country, publication dates, study design, location), objectives, inclusion/exclusion criteria, major findings related to breastfeeding practices and potential solutions suggested by authors of the evidence gathered. The analysis and summary of gray literature were recorded in the same way as it was done for primary studies. Data extraction form is given in Supplementary File.

### 2.3. Data analysis

Challenges and potential solutions were identified from each study and from the gray literature were listed and categorized under one or more suitable headings based on the WHO building blocks for health systems (32). This framework describes health systems in terms of six core components or “building blocks” viz (i) leadership/governance, (ii) healthcare financing, (iii) health workforce, (iv) access to essential medicines, (v) service delivery, and (vi) health information systems, and has been a popular framework used for health systems research over the years. Studies conducted for assessing if the framework served the purpose for strengthening public health facilities in resource constrained settings have reported in its favor (33). We identified bottlenecks for each building block and they were tabulated as challenges with their potential solutions. Following working definitions were used:

- Bottleneck: any factor that hinders or limits the ability of a health system to deliver the care as per recommended guidelines and therefore poses a barrier to delivering high quality maternal and newborn care to improve health outcomes.
- Leadership/governance: refers to the management and governance of the health system, including policy development, regulation, and accountability, effectiveness of regulatory frameworks, new organizational practices and policies, capacity to assemble and manage resources.
- Healthcare financing: refers to the availability and use of financial resources to support the health system; efficient and effective healthcare financing system, effective budget consumption, ensuring financial sustainability, and cost-effective interventions.
- Health workforce: refers to the availability, distribution, and quality of health workers; number of health workers, their training and qualifications, motivation, appropriate and timely feedback.
- Access to essential medicines: refers to the availability and affordability of essential medicines, vaccines, and technologies; availability and affordability of essential medicines, the quality of medicines and technologies, effectiveness of procurement and supply chain management, networking with the external environment.
- Service Delivery: refers to the provision of essential health services to individuals and populations.; on-time services, mode of communication suitable to patients, and coverage of essential health services.
- Health information systems: refers to the availability and use of reliable health information to support decision-making, planning, and management; availability of health information technology, up-to-date and appropriate guidelines and protocols.

## 3. Results

### 3.1. Study selection

**Figure 1:**
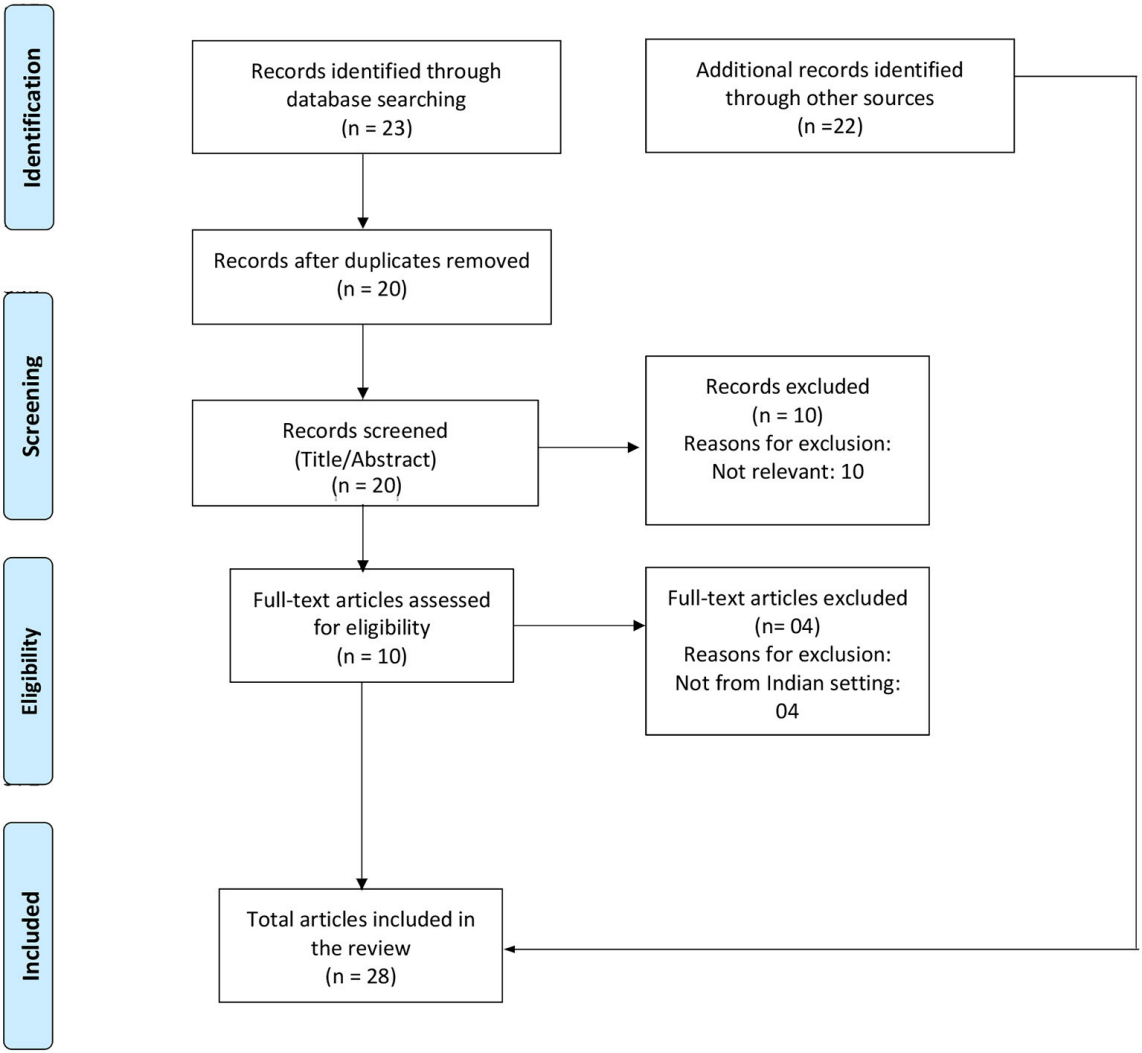
PRISMA flow chart showing the selection and inclusion of the studies in the review.

The database search generated 23 articles. After removing duplicates (*n* = 3) and irrelevant articles (*n* = 14), 6 articles were shortlisted. After adding the resources from gray literature (*n* = 22), a total of 28 documents were included in the review. The PRISMA flow diagram shows the details of the selection of the documents (Figure 1).

### 3.2. Study characteristics

The 28 documents selected included open letters (*n* = 5), original research articles (*n* = 4), press releases and news briefs (*n* = 3), guidance documents (*n* = 3), professional bodies' recommendations (*n* = 3), narrative/expert reviews (*n* = 2), commentaries (*n* = 2), updates and advisories (*n* = 2), position papers and alerts (*n* = 2), case report (*n* = 1), and clinical practice guidelines (*n* = 1). The included articles and their main findings are given in Supplementary Table 2.

### 3.3. Challenges to implementing recommended breastfeeding practices in institutionalized recently delivered mothers, and reported solutions

The primary challenge that surfaced from the study findings was the lack of accurate information on best practices for breastfeeding during a pandemic. This included the absence of standard guidelines, confusion among healthcare workers, and inadequate materials for educating and promoting breastfeeding during COVID-19. Most of the challenges identified were categorized under health service delivery, health workforce, and health management information systems. Specifically, 39.3% of the documents addressed challenges related to leadership and governance, while 35.7% addressed challenges related to health service delivery. In addition, challenges pertaining to the health workforce domain were found in approximately 32.1% of the documents, while 21.4% of the identified challenges fell under the health information system domain. Finally, around 17.9% and 7.1% of the documents included challenges related to the essential medicines and health financing domains, respectively (Table 1).

**Table 1:** Distribution of challenges under the WHO building blocks.

**Health system building block**	**Number of challenges identified**	Number of studies/ documents^*^(*N* = 28)
Leadership and governance	2	11 (39.3)
Health financing	2	2 (7.1)
Health workforce	5	9 (32.1)
Essential medical products and technologies	2	5 (17.9)
Health service delivery	8	10 (35.7)
Health information system	5	6 (21.4)

* The percentage will not be equal to 100% as the challenges are addressed in more than one document.

#### 3.3.1. Leadership and governance

In the initial days, guidelines for breastfeeding during COVID-19 were not in place which led to poor decision-making and planning, and a lot of confusion and misinformation among the healthcare delivery staff as well as the mother and her family (34–38). For example, some guidelines recommended that mothers with COVID-19 should be separated from their infants (38–40). This could have contributed to restriction of skin-to-skin contact and rooming-in in recently delivered mother-newborn dyads. Timely issuance of guidelines/notifications to healthcare providers in all the institutions and case-to-case based decision making on separating mothers and infants were some of the solutions in the literature to overcome this challenge (34, 40–45).

#### 3.3.2. Healthcare financing

Documents reported about the inadequacy of funds for scaling up breastfeeding initiation both in terms of deploying dedicated human resource and logistics (42, 44). For example, availability of donor milk or expressing milk were some of the measures mentioned in the guidelines when direct breastfeeding was not possible. However, maintenance and transportation of donor milk and equipment like breast pumps required additional funding support which was not adequately available. Provision of separate funds for breastfeeding initiation and scale up in hospitals was a probable solution suggested (42, 44).

#### 3.3.3. Health workforce

Health workforce challenges primarily included shortage of health workers, lack of training in breastfeeding support and supervision in the hospitals (36, 37). Many healthcare workers were diverted to COVID-19 management tasks including contact tracing, laboratory testing, triaging and emergency care, leading to disruption of routine healthcare services (42). Additionally, there was a lot of confusion regarding breastfeeding practices during the pandemic. In such situation, health workers were unable to impart the usual counseling and support to mothers regarding breastfeeding (46). The documents recommended that responsive management practices be adopted for improving the workforce situation both in terms of quantity and quality by expeditiously recruiting and training human resources for health and strengthening their knowledge through webinars, online training, etc (36, 39, 42, 46–48).

#### 3.3.4. Access to essential medicines, medical products, and technology

Lack of essential medicines and equipment was another set of challenges observed during the pandemic. Supply and logistics chain was disrupted; there was constant shortage of personal protective equipment (PPEs) and other essential medicines and equipment. Challenges were identified at two levels i.e., the facilities being historically under-resourced and procurement being relatively slow (36, 39). These being systemic challenges that may not be amenable to immediate correction, the reports suggested to focus on improving patient awareness and hospital staff for strengthened IPC for optimizing the need for resource reinforcement (36, 40, 42, 48, 49).

#### 3.3.5. Health service delivery

Service delivery was affected mainly due to disruption in the availability and quality of breastfeeding support. Physical and logistical constraints like lack of space to monitor mothers during breastfeeding and lack of lactation counseling, poor quality of care issues and poor implementation of skin-to-skin contact and possibility of iatrogenic transmission of COVID-19 infection were some of the challenges under this domain (36, 38, 39, 43, 50). The guidelines mandated delivery of basic psychosocial and lactating support to mothers, training to providers and family members regarding breastfeeding support and continued SSC to streamline breastfeeding practices (36, 42–45, 51).

#### 3.3.6. Health information system

A major challenge was the lack of information on breastfeeding initiation and practices in the health management information system (36). Community ownership and partnership related challenges included poor awareness among public regarding exclusive breastfeeding and correct breastfeeding techniques, fear of contracting infection and sociocultural barriers (36, 38, 50, 52). Subsequently, there was a lot of misinformation which led to stress and anxiety among mothers and other family members (36, 43). Awareness campaigns and workshops through videos, infographics, live chats and helplines by professionals and health workers was one potential solution to deal with this challenge (35, 36, 47). It was also suggested that systems should be put in place to ensure that data related to breastfeeding practices was adequately recorded in the hospital's health information database for informing subsequent action (50).

The details of the challenges and suggested solutions for these challenges have been listed in Table 2.

**Table 2:** Challenges to optimal breastfeeding practices and the potential solutions.

**Health system building block**	**Sub-category**	**Significant challenges**	**Suggested solutions**
Leadership and Governance	Policy and guidelines	Absence of national policies/strategies and/or service guidelines for breastfeeding during the COVID-19 pandemic (34–38)	Issuance of guidelines/notifications to healthcare providers in both government and private hospitals for breastfeeding practices in institutionalized mothers (34, 41)
		Mother infant separation into different wards or within the same room based on suspected/confirmed COVID-19 (38–40)	Decision on separation to be made on a case-to-case basis and clinical condition of mother. Rooming-in should be preferred even if the mother and her infant have suspected, probable or confirmed SARS-Co-V2 infection (40, 42–45, 53)
Health financing	Funding	Funding limited or not available for implementation and scale-up. Expression of breast milk and transport requires additional equipment (e.g., breast pumps) which may require funding support (31, 34).	Provision on separate funds for breastfeeding initiation and scale up in hospitals (31, 34).
	Out of pocket costs	Burden of out-of-pocket expenditures by caregivers (34)	
Health Workforce	Number, competence, distribution of health worker	Shortage of competent health workers and poor distribution of properly trained personnel authorized to provide breastfeeding support (36, 37) Diversion of existing health personnel to COVID wards and emergency care disrupting routine health care services (31) Limited staff for carrying out ICPs (36, 38, 39)	Improving workforce in terms of numbers and by training of existing personnel in breastfeeding support (31). Streamlining and ensuring infection control practices in the mother and newborn wards through appointment of new staff and training of existing staff (36, 37).
	Training	Lack of training of health workers on breastfeeding support to mothers during a pandemic (47)	Sensitization of Covid-19 disease managers and training of the health care workers looking after COVID-19 positive mothers in initiation of breastfeeding and supporting mother and family to breastfeed should be included as a part of management strategy of the pandemic. Training can be carried out by subject experts in batches and on a periodic basis to ensure all rotating staff are also covered (29, 45). Strengthening of knowledge regarding common neonatal issues, especially breastfeeding, among all pediatricians and obstetricians (36) Sensitisation of officials and frontline functionaries [such as community health workers] on COVID-19 and infant feeding may be conducted through webinars (29, 45). Online training for safe breastfeeding to Anganwadi supervisors in rural parts of the country can be explored in the event of a pandemic (29, 45).
	Mentorship and supervision	Lack of mentorship and supervision mechanisms for breastfeeding (46)	Periodic collection and audit of data on breastfeeding initiation and continuation needs to be put in place to improve supervision mechanisms (40, 46).
Essential medical products and technologies	Resources	Unavailability of resources and supplies needed for donor human milk	Expressed breast milk and donor milk needs to be provided where mother and baby are separated. For this, proper systems should be set in place (40) Greater vigilance of donor screening procedures needs to be followed. Symptomatic/at-risk donors should be avoided (36) Strict hygiene procedures need to be followed while supporting expression, transportation, and handling of milk (40) A social media group of lactating mothers can be developed to motivate them to donate milk and methods may be devised to pick donated milk from homes (45). Funding of research on donor breastmilk and COVID-19 is necessary for future planning (31).
	Procurement	Poor procurement and supply chain logistics for Infection control practices (ICPs) (36, 39)	Hospitals should have isolation zones which should include outpatient, ward, ICU, labor rooms and operation theaters demarcated for COVID-19 infected women. This needs to be achieved primarily by reorganization of exiting infrastructural setup (49) Mothers should have access to appropriate guidance and logistics like masks, water and soap etc. to practice respiratory hygiene in the health facility and at home (40, 42, 49, 53) Individual dedicated breast pumps should be used. After each pumping session, all parts that come into contact with breast milk should be thoroughly washed and the entire pump should be appropriately disinfected as per the manufacturer's instructions (42) All surfaces in the breastfeeding room should be routinely disinfected and cleaned with readily available products (40)
Health service delivery	Physical and logistical constraints	Lack of space and logistical constraints related to support and monitor mothers during breastfeeding (38)	Counseling for lactating mothers is critical for maintaining breastmilk supply from mother to the baby and should be ensured throughout (42, 53) Restriction of the word “COVID” in both verbal and written statements given to patients alleviates anxiety (43)
		Lack of lactation counseling support (38, 39)	Basic psychosocial support and practical feeding support needs to be provided to manage common breastfeeding difficulties during the post-natal period and should be available in all health facilities (43, 53) Introduction of music and religious book reading sessions can make mothers feel relaxed (31). Providers or family members (with personal protection equipment) should provide support in breastfeeding and caring for new-borns. This training should be reinforced once before discharge and such reinforcement can be continued in the community by health workers (36).
	Quality	Poor quality of care issues and poor implementation of skin-to-skin contact (Kangaroo mother care) (36, 38)	Continued skin to skin contact to be mandatorily practiced (40, 42, 44, 45, 51, 53)
		Possibility of iatrogenic transmission of COVID-19 infection (38)	
		Higher numbers of operative deliveries (50)	
	Follow up	Lack of follow-up of breastfeeding practices after discharge (43)		
	Availability and delivery	Shortage of staff to handle the collection and transfer of expressed mother's milk (36, 50) Poor access to services at the health facility (38)	
Health Information system	Availability of information	Lack of information, records and data on breastfeeding practices and initiation of breastfeeding (36)	Ensuring data related to breastfeeding practices is recorded in hospital health information systems (36)
	Knowledge and Awareness	Lack of awareness and knowledge of benefits of exclusive breastfeeding and correct breast feeding techniques (38) Fear of transmitting COVID-19 to the infant (36, 38) Use of breast milk substitutes (38, 50) Unpredictable, uncertain, serious nature of COVID19, along with misinformation and social isolation contributes to stress and mental morbidity (36, 43)	Information on optimal breastfeeding practices may be disseminated through platforms like calls, live chats, and dedicated helplines (36) Awareness on breastfeeding can be generated through videos and infographics (36) Obstetricians may be involved in awareness programs related to breastfeeding (47, 50) Lactation counselors can use microphone to conduct group counseling while maintaining adequate distance (50) A well -structured seminar/webinar by an expert would be a better way to share crucial information/guidelines where further doubts can be cleared (35) In-person workshop for lactating mother may be conducted (35, 50) One to one virtual consultation may be done to overcome difficulties of feeding during pandemic by volunteer lactation consultants (50) Launch of applications to provide access to skilled counseling support for breastfeeding mothers may be explored (50)
	Socio-cultural barriers	Socio-cultural barriers to the practice of KMC (36) Distribution and donation of breast milk substitutes (38, 52) Administration of alternative supplementary foods (38)	
	Access	Travel restrictions in the form of limited public transport and restriction on private vehicle use. Reduced outpatient clinics (36)	

## 4. Discussion

This review highlights the issues surrounding breastfeeding practices in institutionalized recently delivered mother-newborn dyads in India during the COVID-19 pandemic. Separation of mother and the newborn immediately after birth, inadequate human resources and resources for infection control practices, besides unavailability of supportive guidelines and protocols made implementation of recommended breastfeeding practices difficult. Infrastructural gaps e.g., donor milk storage and milk transport support, resulted in use of alternative feeding methods like formula milk. Solutions suggested by the evidence primarily focused on timely issuance of standard guidelines and protocols, awareness generation, lactation support and training of health care professionals regarding methods and importance of optimal breastfeeding practices. Use of telemedicine technology and group sessions were suggested by few articles as alternative strategies. We observed that institutions could identify work around solutions for every challenge they faced though many of these could be temporary. Thus, we are encouraged to infer that there is hope to work toward improved health system preparedness for resilience during health emergencies.

In hospital settings, it has been reported that separation of the mother and the baby at birth and delay in initiating breastfeeding during the COVID-19 pandemic had lasting adverse effect on breast milk feeding outcomes even in well-resourced contexts (54). In their review, Spatz et al. go on further to report that lack of skin-to-skin contact, inadequate lactation support, and insufficient care and education for mothers served as additional challenges to breastfeeding during the pandemic (55). Kotlar et al. also revealed that weak healthcare infrastructure and resources negatively impacted maternal outcomes and breastfeeding practices (48). These were the major challenges identified in our review as well. In our review, bottlenecks were noted for all the six building blocks of health systems with the maximum number of challenges being identified in the blocks of health service delivery (*n* = 8), health workforce (*n* = 5) and health management information system (*n* = 5). Of the six building blocks, challenges in the leadership and governance block appeared in the maximum number of documents that we reviewed (in 11 of 28; 39.3%). Two challenges were noted in the leadership and governance block i.e., unavailability of guidelines and separation of the mother and the newborn at birth. Similarly, even if just two articles mentioned about challenges with health financing, these were quite serious ones—lack of resources for institutional infrastructure and inadequate cover for out-of-pocket expenditure. Thus, these two blocks could have offered significant operational and structural challenges to surge planning during the pandemic. In 2015, Dickson et in follow-through to the Every Newborn Action Plan and the Strategy for Ending Preventable Maternal Mortality (EPMM), did an in-depth analysis of health systems bottlenecks for scaling up quality interventions for newborn care in 12 low-and-middle-income countries in the Indian sub-continent and in Africa (56). They found that the major health system bottlenecks for breastfeeding were of health financing, health service delivery, health workforce, and essential medical products and technologies. Our findings concur with this. We, hence, observe that the pandemic uncovered inherent gaps in health systems for breastfeeding and if these gaps were plugged with contextual interventions, then the system could become more resilient.

We believe that the absence of clear, evidence-based guidelines in the local or regional context (block: leadership and governance) had a central role to play in the disruptions that the pandemic caused to institutional breastfeeding practices. Countries across the globe followed different approaches and protocols at the beginning of the pandemic. There were several modifications to international recommendations for breastfeeding at the hospital during the first year of the COVID-19 pandemic (57–59). Early in the pandemic, researchers through a collaborative effort conducted a review of 33 countries to assess the alignment of their national guidelines for maternal and child health practices during the pandemic with WHO recommendations. They found that none of these recommended all the aspects of the WHO guidance (29). India, like several other countries, had missed on many WHO recommended practices in the initial course of the pandemic (29, 30). Practices that had been overlooked initially were that of rooming-in, early initiation of breastfeeding and direct breastfeeding with infection control measures. However, in due course of time, India improved its alignment score by six points, as reported in a study by Gibble et al., where 101 countries' guidelines were scored based on their alignment with WHO recommendations (30). We, thus, appreciate the leadership that WHO recommendations could play in motivating member states in sustaining breastfeeding practices during public health emergencies despite resource constraints, and the intentionality of countries to make efforts for safeguarding recommended breastfeeding practices.

A system with a strong resource base is likely to be more resilient to disruptions. India's health system is impeded by years of under-investment.

India allocates 1.3% of the GDP to health which is much less than other countries where the average share is 3.6–7% (60). Furthermore, the country does not have a dedicated workforce for supporting breastfeeding practices, even in its most apex healthcare institutions. Consequently, the inherent fragility of the institutional practices for breastfeeding was obvious in the initial days of the pandemic. Nevertheless, it was also evident that the country's health system has the potential to learn and adapt. Putting the needful systems in place and consistently updating the existing capacity is needed to make the breastfeeding services strong and resilient to collateral damage from a pandemic. It is important to highlight the fact that recommendations against breastfeeding should not be made without sufficient evidence that they are necessary. At the same time, mass communication channels can be used to increase awareness regarding early initiation and continued exclusive breastfeeding among the general public to reduce myths and misinformation during pandemics.

### 4.1. Limitations

The major limitations of this review are that we did not take into account the breastfeeding practices after discharge, at home and thereafter. Understanding that the pandemic situation was exigent, we also acknowledge that available (published) literature may not be comprehensive. Further, we did not include articles from countries other than India. So, there is a likelihood of potentially relatable learnings that remain to be enumerated beyond that highlighted in this manuscript. While our analysis highlights that the challenges to breastfeeding existed across all the six pillars, these were complex and systemic. It is inadequately understood as to how populations and health systems respond to public health emergencies given their unique lived experiences, risk perceptions and preparedness (61). Nevertheless, breastfeeding practices continue to be amenable to socio-cultural influences, at least in resource constrained settings (62–64). The WHO building blocks for health systems does not allow for commentary on inter-dependencies between the blocks and with due consideration of the socio-cultural context; we accept this as a major limitation to our study since we have weaved our findings around these building blocks. The framework's inadequacies were obvious during the pandemic when global public health approaches demanded systems thinking with due attention to complexities. It is in the pandemic context that governance was identified as the major “under-pinning” of health system strengthening that cuts across all the building blocks (65).

## 5. Conclusion

The COVID-19 pandemic had a significant impact on the breastfeeding practices among institutionalized recently delivered mothers. Health service delivery, health workforce and health management information system were the most commonly affected areas. Nevertheless, the pandemic has provided rich learning opportunities for health system strengthening. A roadmap should be in place as a result from these learnings and experiences. Establishing learning loops at individual, team and at organizational levels can facilitate the adaptation of best practices from within and beyond institutional settings, and help optimize breastfeeding practices during future pandemics.

## Data availability statement

The original contributions presented in the study are included in the article/Supplementary material, further inquiries can be directed to the corresponding authors.

## Author contributions

ND and DS: tool development, literature search, data acquisition, data analysis, manuscript preparation, and manuscript editing. PP, RM, and SN: data acquisition, manuscript editing, and manuscript review. AMa: concepts, design, definition of intellectual content, tool development, manuscript editing, manuscript review, and guarantor. AMo: concepts, design, definition of intellectual content, tool development, data acquisition, data analysis, manuscript editing, and guarantor. All authors contributed to drafting of the article and approved the submitted version.
