# Supplementary material for: Implementing recommended breastfeeding practices in healthcare facilities in India during the COVID-19 pandemic: a scoping review of health system bottlenecks and potential solutions
Source: Front Nutr. 2023 Jul 31;10:1142089. doi: 10.3389/fnut.2023.1142089 (PMC10423995; doi:10.3389/fnut.2023.1142089)
Supplement: Supplementary file 1 [file Data_Sheet_1.PDF]

Table 1: Detailed Search Strategy

Search Block	Terms	Result
**PubMed**		
#1 (Population)	(("mother*"[Title/Abstract] OR "female*"[Title/Abstract] OR "woman"[Title/Abstract] OR "Women"[Title/Abstract] OR "Female"[MeSH Terms] OR "Women"[MeSH Terms] OR "neonate*"[Title/Abstract] OR "newborn*"[Title/Abstract] OR "infant*"[Title/Abstract] OR "Infant"[MeSH Terms] OR "infant, newborn"[MeSH Terms] OR "recently delivered"[Title/Abstract] OR "nursing mother*"[Title/Abstract])	10,403,623
#2 (Exposure)	("Pandemics"[MeSH Terms] OR "pandemic*"[Title/Abstract] OR "COVID-19"[MeSH Terms] OR "COVID-19"[Title/Abstract] OR "Coronavirus"[MeSH Terms] OR "Coronavirus"[Title/Abstract] OR "SARS-CoV-2"[MeSH Terms] OR "SARS-CoV-2"[Title/Abstract] OR "severe acute respiratory syndrome coronavirus 2"[Title/Abstract]	290,811
#3 Outcome	("Breast Feeding"[MeSH Terms] OR "Breast Feeding"[Title/Abstract] OR "breastfeed*"[Title/Abstract] OR "Lactation"[MeSH Terms] OR "lactat*"[Title/Abstract])	241,631
#4 Location	("institutional*"[All Fields] OR "hospitali*"[All Fields] OR "inpatient"[All Fields] OR "in-patient"[All Fields] OR "admitted"[All Fields] OR "maternity ward"[All Fields] OR "nursery"[All Fields]))	966,714
#5 (Setting)	"india"[Title/Abstract] OR "indian"[Title/Abstract] OR "india"[MeSH Terms]	210,250
#6 (Results)	#1 AND #2 AND #3 AND #4 AND #5	20
**Web of Science**		
#1	TI= (mother* OR female* OR woman OR Women OR neonate* OR newborn* OR infant* OR "recently delivered" OR "nursing mother*") OR AB= (mother* OR female* OR woman OR Women OR neonate* OR newborn* OR infant* OR "recently delivered" OR "nursing mother*")	2,603,243

#2	TI= (pandemic* OR "COVID-19" OR Coronavirus OR "SARS-CoV-2" OR "severe acute respiratory syndrome coronavirus 2") OR AB= ("pandemic*" OR "COVID-19" OR Coronavirus OR "SARS-CoV-2" OR "severe acute respiratory syndrome coronavirus 2")	261,190
#3	TI= ("Breast Feeding" OR breastfeed* OR lactat*) OR AB= ("Breast Feeding" OR breastfeed* OR lactat*)	189,441
#4	TS= (institutional* OR hospitali* OR inpatient OR "in-patient" OR "admitted" OR "maternity ward" OR nursery)	782,963
#5	TI= ("india" OR "indian")	26,499
#6	#1 AND #2 AND #3 AND #4 AND #5	03

Table 2: Summary of all the articles included

S.No.	Title	Authors	Type of article	Year	Country	Possible Challenges to breastfeeding identified	Possible solutions / mitigation strategies suggested by authors
1.	Role of human milk banks amid COVID 19: perspective from a milk bank in India(1)	Maheshwar Bhasin, Sushma Nangia and Srishti Goel	Commentary	2020	India	Separation of mother and child. Feasibility and capability of donor human milk banks to sustain babies as there is shortage of raw milk to donor milk banks. There is paucity of literature explaining role of human milk banks in promoting breastfeeding and providing donor milk as gap support to isolated infants in NICU and PICU. Lack of lactation counsellor. Economic feasibility of “high level of disinfection” to clean plasticwares in milk banks, single use disposable bottles in resource limited environment.	Donor milk has been suggested in scenarios where mother and baby are separated.

2.	Concerns on separation of babies from Mothers and bottle feeding(2)	Dr. Arun Gupta	Letter to the Government	2020	India	Separation of mother and child due to COVID-19	• Continued skin to skin contact and initiation of bf within first 60 minutes after the birth with precautionary measures of respiratory hygiene<br>• Hand hygiene while carrying the newborn.<br>• Decision may be taken on case to case basis depending on the clinical condition of mother and newborn.<br>• During breastfeeding by mother with COVID -19, following precautions are necessary<br>○ Mother should wear a mask and practice respiratory hygiene.<br>○ Mother should wash hands (for 40 seconds) before each feeding session.<br>○ Surfaces should be routinely disinfected and cleaned.<br>• If a woman with COVID -19 is too unwell to breastfeed, she can be supported to safely express milk and by using donor human milk with following precautions<br>○ Practice hand hygiene<br>○ A dedicated breast pump should be used.<br>○ After each pumping session, all parts that come into contact with breast milk should be thoroughly washed and the entire pump should be appropriately disinfected<br>○ Expressed breast milk should be fed to the newborn by a healthy caregiver"

3.	The ration support containing commercial baby foods may cause more harm than good to health of babies(3)	BPNI	Press Release	2020	India	Infant formula being not sterile product (acc to WHO) were distributed by NGO among mothers as a substitute for bf.<br><br>Donation of infant formula is not sustainable and can lead to diluting the milk powder, which will perpetuate undernutrition.	In light of this issue BPNI demanded from the govt:<br>• to issue notification to states, districts and police that "free distribution or donations of baby foods for children under two is harmful and prohibited".<br>• The information should be disseminated to the nation through MoHFW's daily briefings.<br>• Establishment of a criteria for handing over a substitute for any baby after assessment of individual baby is carried out. 4. MOHFW may also include in its notification "Supply of provisions to meet the needs of infants/ small children.<br>• Counselling for lactating mothers<br>• District Magistrates and State Planners for Covid19 may use their powers to stop free supplies and donations of infant formula in ration supplies.
4.	Clinical management of severe acute respiratory infection (SARI) when COVID-19 disease is suspected(4)	WHO	Interim Guidance	2020		• All recently pregnant women with COVID-19 or who have recovered from COVID-19 should be provided with necessary information and counselling on safe infant feeding and appropriate IPC measures to prevent COVID-19 transmission.<br>• Infants born to mothers with suspected, probable or confirmed COVID-19 infection, should be fed according to standard infant feeding guidelines, while applying necessary precautions for IPC.<br>• As with all confirmed or suspected COVID-19 cases, symptomatic mothers who are breastfeeding or practising skin-to-skin contact or kangaroo mother care should practise respiratory hygiene, perform hand hygiene before and after contact with the child, and routinely clean and disinfect surfaces which the symptomatic mother has been in contact with.<br>• Breastfeeding counselling, basic psychosocial support and practical feeding support should be provided to all pregnant women and mothers with infants and young children.<br>• In situations when severe illness in a mother due to COVID-19 or other complications prevent her from caring for her infant or prevent her from continuing direct breastfeeding, mothers should be encouraged and supported to express milk, and safely provide breastmilk to the infant, while applying appropriate IPC measures.	

						• Mothers and infants should be enabled to remain together and practise skin-to-skin contact, KMC and to remain together and to practise rooming-in throughout the day and night, especially immediately after birth during establishment of breastfeeding.	
5.	Why I Can't Breastfeed My New-born Baby? Psychosocial Dilemma of a COVID-Positive Post-LSCS Mother(5)	Sanjeev Kumar et al	Case Report	2020	India	Stress, depression, irritability, insomnia, fear, confusion, anger, frustration, boredom, and stigma in the mother who was unable to breastfeed her newborn.<br>Unpredictable, uncertain, serious nature of COVID19, along with misinformation and social isolation contributes to stress and mental morbidity.	• To avoid stigma, it is advised to restrict the word "COVID" in both verbal and written statements given to patients.<br>• A COVID-positive asymptomatic or symptomatic mother should be allowed to breastfeed her baby with strict infection control measures.
6.	COVID-19 and Breastfeeding Information Update(6)	BPNI	Update	2020	India	There are concerns about practicing skin-to-skin touch, breastfeeding and optimal infant and young child feeding in the SARS-CoV-2 virus suspected, probable or confirmed mothers.	• Comprehensive lactation support and counselling.<br>• Mothers with suspected, probable or confirmed SARS-Co-V2 infection should practice optimal infant and young child feeding practices.<br>• Necessary precautions for IPC such as hand washing before touching the baby, face mask, cleaning the surfaces and physical distancing should apply.<br>• Rooming-in should be practiced throughout the day and night, while observing appropriate IPC measures.<br>• Expressed milk should be given with a clean cup and/or spoon by a healthy care giver.<br>• In a situation when the mother is unable to breastfeed or express breastmilk, wet nursing, donor human milk, relactation or appropriate breastmilk substitutes should be used.<br>• Health care facilities should not accept any donations of free or subsidized supplies of breastmilk substitutes from any source. The Infant Milk Substitutes

							Feeding Bottles, and Infant Foods (Regulation of Production, Supply and Distribution) Act 1992, and Amendment Act 2003 should be enforced effectively.<br>• Any promotion of breastmilk substitutes, feeding in the health facilities and public should not be allowed.<br>• Free and subsidised supply /donations of infant milk substitutes/infant foods for children under the age 2 should not be allowed.<br>• Skilled breastfeeding counselling and support, basic psychosocial support and practical feeding support to manage common breastfeeding difficulties.<br>• Sensitization of Covid-19 disease managers and training of the health care workers looking after SARS-Co-V2 positive parturient mothers should be a part of management strategy of Covid-19 pandemic.<br>• Mothers should have access to appropriate guidance and logistics like masks, water and soap etc. to practice respiratory hygiene in the health facility and at home.<br>• Efforts to protect, promote and support breastfeeding should be further strengthened to boost immunity of infants and young children.<br>• Mothers should be counselled/advised to continue breastfeeding, if the infant or young child becomes sick with suspected, probable, or confirmed COVID-19 or any other illness.

7.	WHO EMRO Breastfeeding advice during the COVID-19 outbreak (Hindi)(7)	WHO	Advisory	2020	India	Contents of advisory:<br>• Wearing mask during breastfeeding.<br>• Maintain hand hygiene before and after breastfeeding<br>• Sanitize and disinfect the surfaces which are in contact frequently.<br>• If the mother is already COVID positive: 1. Practice expressing milk if too unwell or if direct breastfeeding is not possible 2. In extreme cases, another woman can be identified for breastfeeding 3. Milk from milk banks can also be utilized during these times Upon being well again, one can consider relactation with proper support and counselling. For health care institutions and workers: 1. Carton milk, bottle milk and pacifiers should not be used in maternity and child homes 2. Mother and baby should be kept together immediately after the birth. They should be kept in the same room.<br>• Follow KMC and skin to skin contact. This should be followed even if there is suspicion of COVID-19 infection.<br>• Counselling and psychological support.<br>• Important Guidelines for Infant Feeding: 1. Breastfeeding to be started within 1 hour of birth 2. Exclusive breastfeeding to be followed till 6 months 2. Start supplementary diet at the end of 6 months that is safe and adequate 3. Breastfeeding to be practiced till 2 years and more if possible.	
8.	When Separation is not the Answer: Breastfeeding Mothers and Infants affected by COVID-19(8)	Cecília Tomori et al	Commentary	2020		1.The sequelae of COVID-19-related separation and insufficient bf is not adequately mapped. 2. Separation limits breastfeeding's protection against infectious disease. 3. Immediate postpartum separation introduces significant risk of secondary iatrogenic harms due to disruption of breastfeeding, particularly in communities already facing marginalization, poor health, and structural barriers to care prior to the COVID-19 pandemic. 4. Panic buying of infant formula after emergency measure declarations have resulted in shortages.<br>5. Families without access to appropriate breastmilk substitutes may be forced to resort to even more risky alternatives such as unmodified cow's milk or early use of solid foods. 6. Pandemic being used as a marketing opportunity for infant formula manufacturers that will undermine overall bf and consequently maternal and infant health. 7. Separation does not ensure lower viral	• Consideration of any clinical or public health intervention that may reduce the ability of infants to be exclusively breastfed must be seriously interrogated.<br><br>• Health services must fully consider the short-and-long-term impacts of separating mothers and infants in their policies.

						exposure during hospitalization or afterwards. 8. Shortage of PPE, testing capacity, and staff needed to implement recommended IPC in facilities. 9. Additionally, alternate caregivers who appear healthy may still be sources of exposure. 10. Under high load pandemic conditions, separation may not be a feasible strategy for those mothers and infants who do not require separate care due to severe illness. 11. Lack of bf is associated with increased risk of postpartum haemorrhage, maternal anaemia, and closely spaced births all of which can be life threatening. 12. Women who do not breastfeed also face greater risks of breast and other reproductive cancers, and cardiovascular morbidity and mortality. 13. Current COVID-19 postpartum separation policies may exacerbate the negative effects of chronic unresolved grief and psychological distress associated with these recent and historical traumas. 14. Concerns about severe disease from SARS-CoV-2 in infants appear to be the main driver of guidelines for postpartum separation of mothers and infants. this strategy does not sufficiently account for epidemiological evidence or opportunity costs and underestimates the expected sequelae of separation for PUI/confirmed positive mothers and infants.	
9.	Gaps in beliefs and practices of trained breastfeeding counsellors and lactating mothers facing Covid-19 pandemic in India(9)	BPNI	Research article	2020	India	1. one-third of the study participants did not have correct knowledge about the passage of Covid-19 virus through the placenta and 10% were not aware that Covid-19 virus does not pass through breastmilk. 2. 7% participants even believed that Covid-19 positive mothers should avoid breastfeeding and use infant formula. 3. Perception of mothers of not enough milk and breast problems and anxiety about practicing breastfeeding in view of pandemic. 4. Only 6 (30%) participants	• Need of on-going updating and orientation of these trained professionals with the technical information about the safety and necessity of breastfeeding by the Covid-19 positive mothers<br>• The pregnant and lactating women should be helped with correct information about breastfeeding in Covid-19 situation in local language and using easy to understand print and electronic instruments to address the

						practiced rooming in, 7 (35%) kept the mother and baby in the same room but baby was kept away from mother's bed 7 (35%) kept the baby in another room or in the neonatal unit.	anxiety about practicing breastfeeding in view of Covid-19 pandemic.
10.	Ensuring Exclusive Human Milk Diet for All Babies in COVID-19 Times(10)	Ruchika Chugh Sachdeva et al	Narrative Review	2020	India	1. Suspension of outpatient services, 2. Travel restrictions 3. Staff shortage 4. Mothers get little opportunity to learn to breastfeed or express and feed mothers' milk to their babies due to which, they are resorting to formula feeding at home 5. Due to infrastructure constraints and mandate to transfer COVID-19 positive mothers to separate COVID-19 wards, many facilities are separating COVID-19 positive mothers from their babies 6. Some facilities end up feeding PDHM or formula owing to a shortage of staff to handle the collection and transfer of expressed mother's milk. 6. Mothers and families, and health care workers have fear and confusion around COVID-19 and breastfeeding, along with a sense of heightened stigma about the disease which is impacting access to breastmilk. 7. Fear of transmission by KMC. Rooming-in is difficult as COVID-19 positive mothers are shifted to COVID-19 wards/ facilities while their babies remain in the neonatal units. 8. Many centers have temporarily suspended human milk banking services for reasons such as absenteeism and irregularity of staff due to imposed lockdown. Number of facility-based milk donors has reduced because of earlier discharges and discontinuation of outpatient services. Amid lockdown, home collection of donor milk, and community milk collection has almost stopped. Some mothers may be reluctant to donate fearing donation as a point of exposure. Few hospitals are finding it hard	• Hospitals should provide more information, psychosocial, and technical support to lactating mothers and their families to Providers or family members with PPE should provide support in breastfeeding and caring for newborns.<br>• Facilities have reported adopting various methods to improve lactation in mothers, including introducing music and religious book reading sessions to make mothers feel relaxed; allocating rooms in NICUs for mothers along with regular thermal screening and adherence to strict hygiene measures; arranging for additional breast pumps for all mothers; and information dissemination platforms through calls, live chats, and dedicated helplines.<br>• Facilities where isolation of COVID-19 suspected/ confirmed mother and her baby is possible, rooming-in with direct breastfeeding should take place. Where isolation is not possible, mother should be temporarily separated from baby until she is confirmed negative.<br>• Lactation counselling should be coupled with optimal lactation support to ensure maternal milk supply at the earliest. This reduces the excess demand for PDHM which can be given to those who need it the most.<br>• Greater vigilance and modification of donor screening procedures. CLMC staff are asking additional questions related to symptoms, travel and contact history, and excluding symptomatic / at-risk donors.

						to devote time to milk culture and outsourcing has become difficult for others due to transportation restrictions. To make up for the reduced supply, some units are rationing PDHM to the most vulnerable, small, and sick babies, which may lead to increase in formula feeding for babies without access to maternal milk.	• Strict hygiene procedures are followed while supporting expression, transportation, and handling of milk.<br>• Strengthening of lactation management systems to universalize access to human milk is a key strategy to reduce newborn morbidity and mortality during and beyond the pandemic.<br>• Highlight importance of neonatal nutrition during the pandemic; fund research on breastmilk and COVID-19; facilitate innovations across CLMC processes to improve access to maternal milk and PDHM; equip zonal reference centres to support facilities to adequately prepare for future emergencies; and support the consolidation of the countrywide Human Milk Bank Association of India to facilitate communication, sharing of data and best practices, and evidence generation among milk banks.
11.	Commercial Baby foods during COVID19 Pandemic emergency response(11)	BPNI	Letter	2020	India	• Supply of provisions to meet the needs of infants/ small children<br>• Counselling for lactating mothers, and preventing unnecessary donation, distribution and misuse of baby foods and ensuring provision of counselling to the lactating mothers.<br>• Depending on the assessment of the situation if there is any need to provide infant milk substitutes, it may be used under supervision.	
12.	FOGSI GCPR- Good Clinical Practice recommendation on Pregnancy with COVID-19 Infection(12)	FOGSI GCPR	Recommendations	2020	India	• Arrangements in existing healthcare facilities to manage COVID-19 exposed and infected pregnant women<br>• Hospitals should have isolation zones which should include outpatient, ward, ICU, labour rooms and operation theatres demarcated for COVID-19 infected women.<br>• Newborn care should be practiced as per routine.<br>• Testing is recommended if the mother has COVID-19 infection or if the baby is symptomatic.<br>• Breastfeeding can be given with good hygiene practices.	

13.	Perinatal-Neonatal management of COVID-19 infection(13)	FOGSI, NNF, IAP	Clinical practice guidelines	2020	India	• Stable neonates exposed to COVID-19 infection from mothers or other relatives should be roomed-in with their mothers and be exclusively breastfed.<br>If rooming-in is not possible, the neonate should be fed expressed breast milk of the mother by a nurse or family member who has not been in contact with the mother or other suspected/proven case.<br>• Stable neonates exposed to COVID19 and being roomed-in with their mothers may be discharged together at time of mothers' discharge.<br>• Stable neonates in whom rooming-in is not possible because of the sickness in the mother and are being cared by a trained family member may be discharged from the facility by 24-48 hours of age.
14.	Guidance for Management of Pregnant Women in COVID-19 Pandemic(14)	ICMR NIRRH	Guidance document	2020	India	• Facilities should consider temporarily separating (e.g. separate rooms) the mother who has confirmed COVID-19 or is a PUI, from her baby until the mother's transmission-based precautions are discontinued.<br>• The risks and benefits of temporary separation of the mother from her baby should be discussed with the mother by the healthcare team.<br>• A separate isolation room should be available for the infant while they remain a PUI. The decision to discontinue temporary separation of the mother from her baby should be made on a case-by-case basis Decision should take into account disease severity, illness signs and symptoms, and results of laboratory testing for virus that causes COVID-19, SARS-CoV-2 of mother and neonate.<br>If rooming in of the newborn with his/her ill mother in the same hospital room occurs in accordance with the mother's wishes or is unavoidable due to facility limitations, facilities should consider implementing measures to reduce exposure of the new-born to the virus that causes COVID-19.<br>• Consider using engineering controls like physical barriers (e.g., a curtain between the mother and new-born) and keeping the new-born ≥6 feet away from the ill mother.<br>• If no other healthy adult is present in the room to care for the new-born, a mother who has confirmed COVID-19 or is a PUI should put on a facemask and practice hand hygiene before each feeding. These practices should continue while the mother is on transmission-based precautions in a healthcare facility.<br>• Mother should be encouraged to express their breast milk to establish and maintain milk supply. If possible, a dedicated breast pump should be provided. Prior to expressing breast milk, mothers should practice hand hygiene. After each pumping session, all parts that come into contact with breast milk should be thoroughly washed and the entire pump should be appropriately disinfected as per the manufacturer's instructions. This expressed breast milk should be fed to the new-born by a healthy caregiver.<br>• If a mother and new-born do room-in and the mother wishes to feed at the breast, she should put on a facemask and practice hand hygiene before each feeding.

15.	Infant & young child feeding in the context of covid-19(15)	UNICEF, Global Nutrition cluster, GTAM	Recommendation	2020	-	• Establish safe breastfeeding protocols for infected mothers and policies to avoid widespread distribution and donations of BMS while ensuring that infants under 6 months with no possibility to be breastfed are adequately supported with infant formula.<br>• Mothers should be counselled/ advised to continue breastfeeding should the infant or young child become sick with suspected, probable, or confirmed COVID-19 or any other illness.<br>• As with all probable, confirmed or suspected COVID-19 cases, symptomatic mothers who are breastfeeding or practicing skin-to-skin contact or KMC should practice necessary respiratory hygiene (such as, wear a face mask), including during feeding.<br>• If the mother has respiratory symptoms, it is recommended to use a face mask when near a child, if possible, perform hand hygiene before and after contact with the child, and routinely clean and disinfect surfaces.<br>• If the mother is expressing breast milk with a hand, manual or electric breast pump, she should wash her hands before expressing breastmilk or touching any pump or bottle parts and ensure proper pump cleaning after each use.<br>• The expressed breastmilk should be fed to the child using a clean cup and/or spoon, preferably by a person who has no signs or symptoms of illness.<br>• If the mother is too unwell to breastfeed or express breastmilk or donor milk is unavailable, an appropriate BMS should be provided.<br>• Wherever counselling is being delivered through health and community platforms, physical visits should be minimized and use virtual channels should be encouraged (such as phones, social media and others) to provide information to families on breastfeeding and complementary feeding behaviours at critical times. Basic psychosocial support should be a key component of counselling.<br>• In case of operational service delivery through health and other platforms, ensure integration of recommended hygiene practices into the counselling and support.<br>• Delivering key messages on safe food preparation, feeding, storage as well as importance of safe and palatable drinking water for their young children.<br>• Ensure integration of harmonized messages on safe hygiene into all relevant opportunities and multiple communication channels through the health system to ensure its reach and application.

16.	Call to Action on Strong Warning Labels on Front of Unhealthy Packaged Food to Protect People’s Health(16)	BPNI	Letter	2020	India	1.ICMR recommends that COVID19 positive and suspected mothers may be separated temporarily. 2. 'Infant formula’ or 'powdered baby foods' are being supplied in the rapid response. 3. Distributing infant formula freely to the poor community may undermine breastfeeding, which is a dangerous practice.	• Infants born to mothers with suspected, probable or confirmed COVID-19 infection, should be fed according to standard infant feeding guidelines, while applying necessary precautions for IPC.<br>• In case the mother is sick and unable to breastfeed, her express breastmilk should be given to the infant with a clean cup and/or spoon.<br>• Rooming-in should be practiced throughout the day and night, whether the mother and her infant has suspected, probable or confirmed COVID-19 virus infection.<br>• Standard infant feeding guidelines include initiating bf within 1 hour of birth, exclusive breastfeeding for 6 months with timely introduction of adequate, safe and properly fed complementary foods at age 6 months, while continuing breastfeeding up to 2 years of age or beyond.<br>• In a situation when the mother is unable to breastfeed or express breastmilk, relactation, wet nursing, donor human milk or appropriate BMS should be used.<br>• Appropriate precautions should be taken to avoid promotion of BMS, feeding bottles by the health facility and the health care providers.
17.	Guidance note on financial contributions or contributions in-kind from food and beverage companies(17)	UNICEF	Guidance document	2020	India	• In line with existing UNICEF policies, companies that manufacture BMS should continue to be excluded from any CIK, funding engagements or co-branded partnerships.<br>• Donations of BMS, complementary foods and feeding equipment, including bottles and teats, should not be sought or accepted for distribution.<br>• Donation of BMS by manufacturers has been shown to increase the use of substitutes and reduce breastfeeding.	

18.	Breastfeeding in India is disrupted as mothers and babies are separated in the pandemic(18)	Neha Bhatt	Press release	2020	India	1.Stigmatization of bf during the pandemic, as families are adamant to separate mother-newborn. 2. Shortage of health staff to support mothers for breastfeeding 3. Increase in C-section which delayed early initiation of bf and increased formula feeding.	• Creating awareness on breastfeeding through videos and infographics.<br>• Sensitisation of officials to frontline functionaries on covid-19, infant feeding through webinar.<br>• Launch of BPNI app stanpan suraksha to provide access to skilled counselling support for breastfeeding mother.<br>• In-person workshop for lactating mother by BPNI.<br>• One to one virtual consultation to overcome difficulties of feeding during pandemic by volunteer lactation consultants
19.	Commercial baby foods in ration supplies or donations may cause more harm than good to health of babies(19)	BPNI	Action alert	2020	India	Distribution of baby foods like infant formulas to mother for feeding their infants by some philanthropic and other organisations in collaboration with local government.	
20.	Frequently asked questions: Breastfeeding and COVID-19<br>For health care workers(20)	WHO	FAQs-WHO	2020	-	• Active COVID-19 has not to date, been detected in the breastmilk of any mother with confirmed/suspected COVID-19. There is no reason to avoid or stop breastfeeding.<br>• The numerous benefits of skin-to-skin contact and breastfeeding substantially outweigh the potential risks of transmission and illness associated with COVID-19.<br>• While breastfeeding, a mother should still implement appropriate hygiene measures, including wearing a medical mask if available, to reduce the possibility of droplets with COVID-19 being spread to her infant.<br>• Mothers with symptoms of COVID-19 are advised to wear a medical mask, but even if this is not possible, breastfeeding should be continued. Other infection prevention measures, such as washing hands, cleaning surfaces, sneezing or coughing into a tissue are also important.<br>• The best alternatives to breastfeeding a newborn or young infant are: expressed breastmilk, donor human milk, if expressing breastmilk or donor human milk are not feasible or available, then consider: wet-nursing, infant formula milk with measures to ensure that it is feasible, correctly prepared, safe, and sustainable.<br>• A mother can start to breastfeed when she feels well enough to do so. There is no fixed time interval to wait after confirmed/ suspected COVID-19. There is no evidence that breastfeeding changes the clinical course of COVID-19 in a mother.	

						• If a mother is confirmed/suspected to have COVID-19 and is breastfeeding, there is no need to provide a ‘top-up’ with an infant formula milk.<br>• Donations of infant formula milks should not be sought or accepted. If needed, supplies should be purchased based on assessed need.	
21.	Separating Covid Positive Mothers and Babies: Abandoning Breastfeeding(21)	Dr. Arun Gupta	Open letter	2021	India	1. Lack of awareness of accurate information on Covid and breastfeeding and of the scientific evidence that breastmilk carries antibodies to fight Covid-19. 2. Separation as a challenge for initiating exclusive bf. 3. National and global recommendations for Covid positive mothers and breastfeeding not being followed in hospitals. 4. The above situations leading to a favourable environment for baby food companies 5. Possibilities of protection, proper care, and emotional development deprivation among the newborns and unwanted sickness.	BPNI requested:<br>• Issuance of guidelines/ notifications to healthcare providers in both government and private hospitals to not to separate mothers and babies, and continue breastfeeding<br>• To stop the misleading information using mass media on a daily basis. This action may be monitored and action be taken on defaulters.
22.	Government action needed on two fronts(22)	BPNI	Letter	2021	India	Continued violations of the Infant Milk Substitutes Feeding Bottles, and Infant Foods (Regulation of Production, Supply and Distribution) Act 1992, and Amendment Act 2003 by the baby food, feeding bottles, web platforms and e-commerce corporations.	• Awareness to families that mothers who test positive can breastfeed using a mask, and babies at birth be kept in skin to skin contact with the mother.
23.	IBFAN News Brief: Breastfeeding Protection in the Time of Covid-19(23)	IBFAN	News brief	2021	-	• Baby food industries play on the fears and insecurities of parents, and exploit the COVID crisis as an opportunity to increase sales and market share.<br>• Despite laws prohibiting the marketing of baby milk products, leading manufacturers of human milk substitutes (HMS) tell mothers to keep 6 ft way from their baby.<br>• Keeping mother and baby together; the invaluable closeness of skin to skin contact; informing that breastfeeding provides the antibodies against microbes or bacteria a mother may be exposed to<br>• Governments to regulate, monitor, and enforce the marketing of HMS, bottles, and teats.	
24.	Breastfeeding in Coronavirus Disease 2019 (COVID-19): Position Statement of Indian Academy of Pediatrics and Infant and Young Child Feeding Chapter(24)	Ketan Bharadva et al	Position paper	2021	India	• If mother’s milk is not available, wet nursing (with proper safety procedural precautions) may be adopted or pasteurized donor human milk from a standard human milk bank or comprehensive lactation management center can be fed by the caregiver.<br>• The mother and baby should not be separated unless mother is severe ill.<br>• Mother with suspect or confirmed COVID-19 should initiate early and exclusive breastfeeding, skin to skin contact and KMC should be promoted.<br>Skills of manual expression of breast milk by trained staff.<br>• COVID-19 vaccine to be administered to all BF mothers.	

25.	Assessment of knowledge and opinion regarding breastfeeding practices during COVID-19 pandemic among paediatricians and obstetricians in India: an online survey(25)	Sunil Malik et al	Research article	2021	India	Only 57% paediatricians were found to have the adequate level of knowledge regarding correct breastfeeding practices. among paediatricians there is a huge lack of information<br><br>Lack of paediatricians in some of the developing countries.	• There is need to strengthen knowledge regarding common neonatal issues, especially breastfeeding, among all paediatricians and obstetricians.<br>• The involvement of obstetricians in awareness programs related to breastfeeding.<br>• A well -structured seminar/webinar by an expert would be a better way to share crucial information/guidelines where further doubts can be cleared. A sensitisation program on breastfeeding in COVID-19 pandemic is needed on a large scale.<br>• A more rigorous dissemination of information on breastfeeding practices in COVID-19 case management needs to be adopted.
26.	A single-center observational study on clinical features and outcomes of 21 SARS-CoV-2-infected neonates from India(26)	Ruchi Nanavati et al	Research article	2021	India	Findings: Among 198 neonates, COVID-19 was detected in 21 (10.6%).12 were roomed-in and exclusive breastfed (57.1%). The remaining 9 were shifted to NICU in view of respiratory distress. In developing countries artificial feedings have been associated with significant morbidity and mortality.	• The decision of rooming-in should be cautious with emphasis on hygiene precautions.<br>• Further studies are needed to address challenges with rooming-in, breastfeeding, repeat testing of these neonates, and analyze the impact of SARS-CoV-2 infection on the long-term follow-up.
27.	Clinical profile, viral load, management and outcome of neonates born to COVID 19 positive mothers: a tertiary care centre experience from India(27)	Pratima Anand et al	Research article	2021	India	1.Opinions are conflicting regarding rooming-in and direct breastfeeding of a neonate born to COVID-19 positive mother.<br>2. One study reported viral particles of COVID 19 in breast milk; however, it was unclear through which route or source the neonate was infected. 3. Strength and durability of secretory IgA found in breast milk of COVID-19 infected mothers have not been determined yet. 4. Difficulty in Ensuring 3 to 6 feet distance between mother and neonate due to space constraints. 5. Lack of availability of separate staff to provide expressed breast milk	• The scientific brief by WHO recommends breastfeeding to be initiated or continued in suspected as well as confirmed COVID-19 infection.<br>• To consider breast milk as potentially infectious, the presence of replicative COVID-19 virus in cell culture needs to be documented in future studies.<br>• Benefits of breastfeeding vs harms of formula feeding by palade, cost and inability to maintain acceptable hygiene with top feed and hence higher incidence

						to the neonate. 6. Inadequate supply of breast pumps and training of mother in manual expression.	of morbidities and mortality due to top milk should be weighed upon, especially in developing nations.
28.	An update on COVID-19 and pregnancy(28)	Denise J. Jamieson, Sonja A. Rasmussen	Expert review	2022	-	The breastfeeding was considered safe during COVID-19. The study has been conducted which reported that breastfeeding is safe with consistent use of surgical mask, hand hygiene and breast cleaning. The vaccination was encouraged in lactating mother as it is associated with high levels of SARS-CoV-2 antibodies in the breastmilk.	

BPNI: Breastfeeding Promotion Network of India, WHO: World Health Organization, FOGSI: The Federation of Obstetric and Gynaecological Societies of India, NNF: National Neonatology Forum, IAP: Indian Academy of Pediatrics, ICMR: Indian Council of Medical Research, NIRRH: National Institute for Research in Reproductive and Child Health, UNICEF: United Nations Children's Fund
gcPR: good clinical Practice Recommendations, NICU: Neonatal Intensive Care Unit, PICU: Pediatric Intensive Care Unit, IP: Infection, Pvention and Control, PPE: Personal Protective Equipment, PDHM: Pasteurised Donor Human Milk, CLMC: Comprehensive Lactation Management Centers, PUI: Person under investigation, BMS: Breast milk substitutes, KMC: Kangaroo mother care, EBM: expressed breast milk, IMS: Infant Milk Substitutes, IYCF: Infant and Young Child Feeding

References:

1. Bhasin M, Nangia S, Goel S. Role of human milk banks amid COVID 19: perspective from a milk bank in India. International breastfeeding journal. 2020;15. https://doi.org/10.1186/s13006-020-00346-0

2. Arun Gupta (Central Coordinator, BPNI, Delhi, India). Letter to: Dr Harsh Vardhan Concerning Separation of Mothers from Babies during covid19 emergency (Ministry of Health and Family Welfare, Delhi, India). 2020 Apr 17 [cited 2023 Jan 10]. Available from: https://www.bpni.org/wp-content/uploads/2020/04/Letter-to-Dr-Harsh-Vardhan-Concerning-Separation-of-Mothers-from-Babies-during-ovid19-mergency.pdf

3. The ration support containing commercial baby foods may cause more harm than good to health of babies [Internet]. 2022 [cited 2023 Jan 10]. Available from: https://www.bpni.org/wp-content/uploads/2020/04/Press-Release-on-Baby-Food-Ration.pdf

4. World Health O. Clinical management of severe acute respiratory infection (SARI) when COVID-19 disease is suspected. Interim guidance. Pediatr Med Rodz. 2020 May 20;16(1):9–26.

5. Kumar S, Rathore P, Shweta, Krishnapriya V, Haokip N, Thankachan A, et al. Why I Can't Breastfeed My New-born Baby? Psychosocial Dilemma of a COVID-Positive Post-LSCS Mother. Indian Journal of Palliative Care. 2020;26(Suppl 1):S150.

6. BPNI. COVID-19 and Breastfeeding Information Update. 2020. [cited 2023 Jan 10]. Available from: https://www.bpni.org/wp-content/uploads/2020/05/Update-on-COVID-19-and-Breastfeeding.pdf

7. BPNI. WHO EMRO breastfeeding advice during the COVID 19 outbreak Hindi. 2020. [cited 2022 Nov 20]. Available from: https://www.bpni.org/wp-content/uploads/2020/03/WHO-EMRO-breastfeeding-advice-during-the-covid-19-outbreak-Hindi-2020-1.pdf

8. Tomori C, Gribble K, Palmquist AEL, Ververs MT, Gross MS. When separation is not the answer: Breastfeeding mothers and infants affected by COVID-19. Maternal & Child Nutrition. 2020;16(4).

9. BPNI. Covid-19 and Breastfeeding quantitative study. 2020. [cited 2022 Nov 20]. Available from: https://www.bpni.org/wp-content/uploads/2020/11/Covid-19-and-Breastfeeding-a-quantitative-study.pdf

10. Sachdeva RC, Jain S, Mukherjee S, Singh J. Ensuring Exclusive Human Milk Diet for All Babies in COVID-19 Times. Indian Pediatrics. 2020;57(8):730.

11. Arun Gupta (Central Coordinator, BPNI, Delhi, India). Letter to: Dr V Thiruppugazh (JS Policy NDMA New Delhi). Commercial Baby foods during COVID-19 Pandemic emergency response. 2020 Apr 21 [cited 2023 Jan 10]. Available from: https://www.bpni.org/wp-content/uploads/2020/04/Dr-V-Thiruppugazh-JS-Policy-NDMA.pdf

12. FOGSI. Good Clinical Practice recommendations on pregnancy with COVID-19 infection. 2020 [cited 2023 Jan 10]. Available from: https://www.fogsi.org/wp-content/uploads/covid19/fogsi_gcpr_on_pregnancy_with_COVID_19_version_1.pdf

13. Chawla D, Chirla D, Dalwai S, Deorari AK, Ganatra A, Gandhi A, et al. Perinatal-Neonatal Management of COVID-19 Infection Guidelines of the Federation of Obstetric and Gynaecological Societies of India (FOGSI), National Neonatology Forum of India (NNF), and Indian Academy of Pediatrics (IAP). Indian Pediatrics. 2020;57(6):536.

14. ICMR. Guidance for Management of Pregnant Women in COVID-19 Pandemic.2020 [cited 2023 Jan 23]. Available from: https://www.icmr.gov.in/pdf/covid/techdoc/Guidance_for_Management_of_Pregnant_Women_in_COVID19_Pandemic_12042020.pdf

15. UNICEF. IYCF Programming COVID19 Brief. 2020 [cited 2022 Nov 20]. Available from: https://www.unicef.org/media/68281/file/IYCF-Programming-COVID19-Brief.pdf

16. Garg, Suneela (Maulana Azad Medical College, Delhi , India). Letter to: Sh Mansukh Mandaviya (Health Minister, MoHFW, Delhi, India). Call to action on strong Warning Labels on Front of Unhealthy Packaged Food to Protect People’s Health. 2021[cited 2023 Jan 10]. Available from: https://www.bpni.org/wp-content/uploads/2021/09/Letter-to-Health-Minister-Sh.-Mansukh-Mandaviya.pdf

17. COVID-Guidance on financial contributions and contributions in kind by FB. 2020 [cited 2022 Nov 20]. Available from: https://www.nutritioncluster.net/sites/nutritioncluster.com/files/2020-05/COVID-Guidance%20on%20financial%20contributions%20and%20contributions%20in%20kind%20by%20FB.pdf

18. Bhatt N. Breastfeeding in India is disrupted as mothers and babies are separated in the pandemic. BMJ. 2020 Aug 25;370.

19. BPNI. Commercial baby foods in ration supplies or donations may cause more harm than good to health of babies. 2021 [cited 2022 Nov 20]. Available from: https://www.bpni.org/wp-content/uploads/2020/04/Action-Alert-1-COVID-19.pdf

20. WHO. Frequently asked questions: Breastfeeding and COVID-19 for health care workers. 2020 [cited 2022 Nov 20]. Available from: https://cdn.who.int/media/docs/default-source/maternal-health/faqs-breastfeeding-and-covid-19_805d4ce8-2329-4227-9261-695afa68b32c.pdf?sfvrsn=d839e6c0_5&download=true

21. Arun Gupta (Central Coordinator, BPNI, Delhi, India). Letter to: Health ministers of India. Separating Covid Positive Mothers and Babies: Abandoning Breastfeeding. 2021 [cited 2023 Jan 10]. Available from: https://www.bpni.org/wp-content/uploads/2020/04/Dr-V-Thiruppugazh-JS-Policy-NDMA.pdf

22. Nupur Bidla (Director, Advocacy, BPNI). Lwetter to: Dr. Harsh Vardhan (Honble Minister MoHFW, Delhi, India). Government action needed on two fronts. 2021 [cited 2023 Jan 10]. Available from: https://www.bpni.org/wp-content/uploads/2021/05/Dr.-Harsh-Vardhan-Honble-Minister-MoHFW.pdf

23. Sterken E. IBFAN News Brief: Breastfeeding Protection in the Time of Covid-19. Journal of Human Lactation. 2020 Oct 14

24. Bharadva K, Bellad RM, Tiwari S, Somasekar R, Phadke M, Bodhankar U, et al. Breastfeeding in Coronavirus Disease 2019 (COVID-19): Position Statement of Indian Academy of Pediatrics and Infant and Young Child Feeding Chapter. Indian Pediatrics. 2022;59(1):58.

25. Malik S, Joshi P, Gupta PK, Sharma S. Assessment of knowledge and opinion regarding breastfeeding practices during COVID-19 pandemic among paediatricians and obstetricians in India: an online survey. Sudanese Journal of Paediatrics. 2021;21(1):30.

26. Nanavati R, Mascarenhas D, Goyal M, Haribalakrishna A, Nataraj G. A single-center observational study on clinical features and outcomes of 21 SARS-CoV-2-infected neonates from India. European Journal of Pediatrics. 2021;180(6):1895.

27. Anand P, Yadav A, Debata P, Bachani S, Gupta N, Gera R. Clinical profile, viral load, management and outcome of neonates born to COVID 19 positive mothers: a tertiary care centre experience from India. European Journal of Pediatrics. 2021;180(2):547.

28. Jamieson DJ, Rasmussen SA. An update on COVID-19 and pregnancy. American Journal of Obstetrics and Gynecology. 2022;226(2):177.

Data Extraction Form

Data%20Extraction%20form_breastfeeding
